# Isokinetic Identification of Knee Joint Torques before and after Anterior Cruciate Ligament Reconstruction

**DOI:** 10.1371/journal.pone.0144283

**Published:** 2015-12-08

**Authors:** Adam Czaplicki, Marta Jarocka, Jacek Walawski

**Affiliations:** 1 Department of Biomechanics and Computer Science, Faculty of Physical Education and Sport, The Josef Pilsudski University of Physical Education, Biala Podlaska, Poland; 2 Department of Physiotherapy, Faculty of Tourism and Health, The Josef Pilsudski University of Physical Education, Biala Podlaska, Poland; 3 Department of Medical Rehabilitation, Faculty of Tourism and Health, The Josef Pilsudski University of Physical Education, Biala Podlaska, Poland; 4 Department of General Surgery, Orthopaedic Surgery Unit, MSW Hospital, Lublin, Poland; Van Andel Institute, UNITED STATES

## Abstract

The aim of this study was to evaluate the serial change of isokinetic muscle strength of the knees before and after anterior cruciate ligament reconstruction (ACLR) in physically active males and to estimate the time of return to full physical fitness. Extension and flexion torques were measured for the injured and healthy limbs at two angular velocities approximately 1.5 months before the surgery and 3, 6, and 12 months after ACLR. Significant differences (*p* ≤ 0.05) in peak knee extension and flexion torques, hamstring/quadriceps (H/Q) strength ratios, uninvolved/involved limb peak torque ratios, and the normalized work of these muscles between the four stages of rehabilitation were identified. Significant differences between extension peak torques for the injured and healthy limbs were also detected at all stages. The obtained results showed that 12 months of rehabilitation were insufficient for the involved knee joint to recover its strength to the level of strength of the uninvolved knee joint. The results helped to evaluate the progress of the rehabilitation and to implement necessary modifications optimizing the rehabilitation training program. The results of the study may also be used as referential data for physically active males of similar age.

## Introduction

A full recovery following arthroscopic anterior cruciate ligament reconstruction (ACLR) is a priority for both athletes and individuals having an active lifestyle. Since the implementation of accelerated rehabilitation protocols [1,2], numerous researchers [3–8] have been convinced that six months is a sufficient amount of time for the recovery of a knee joint to the level prior to the injury. This view is not commonly accepted [9,10], and the results of some studies have proved that alterations in the kinematics of the reconstructed knee joint [11–13] and deficits in quadriceps strength [14,15] may still occur a year or longer after the reconstruction.

A person can return to sport or intensive physical activity when his/her condition is certified. In order to decide whether or not to issue such a certificate, clinicians apply the standard Lysholm and Gillquist [16] or Tegner [17] scales, as well as using knee arthrometers [18]. Both scales, however, are subjective in nature, and the results of knee laxity measurements may not correlate with the level of the functional performance of the knee joint [19]. The reliability of a medical certificate can be strengthened by common biomechanical tests, which are usually conducted in a direct or indirect way. In the former case, knee torques are measured using dynamometers. In the latter, inverse dynamics based on appropriate biomechanical models is applied [14,20–24].

Torques are very frequently measured in isokinetic conditions due to the fact that this is a traditional method of their assessment [25] which has several applications [2]. This thesis is confirmed in a review paper by Kvist [26], who discusses 34 studies on rehabilitation after ACLR and presents a table illustrating the results of tests assessing the strength and performance of the knee joint muscles. In 16 studies isokinetic tests were used to assess the efficiency of the injured knee. The authors of all of these works except one [3] analyze mean extension and flexion peak torques. Yet, additional information, which is significant from the clinical point of view, may be obtained by examining the torque-position characteristics of these muscles. To the best of our knowledge, an analysis of such characteristics prior to and following ACLR has not been conducted to date.

In the works cited above, internal loads in knee joints after arthroscopic anterior cruciate ligament reconstruction were examined at one or two stages of the patient’s clinical assessment based on the aims of a particular study. For instance, the examinations were performed at least 6 months [20], at least 5 months [14], 5 months or one year [21], half a year or two years [22], and 22 months or 3 years [24] after the operation. In order to determine precisely the time of the subject’s return to full physical fitness, the strength of their knee joint flexors and extensors should be monitored regularly, according to a previously defined schedule; however, due to long research cycles, there is little evidence in the literature that such measurements have been implemented. In addition, the scant research in this domain is based on the analysis of knee joint peak torques in groups which are heterogeneous in terms of gender and age [27,28].

The 4-stage cycle of biomechanical tests proposed by us differs from the methods suggested in the literature in that torque-position characteristics were included into the analysis of the subjects’ progress in rehabilitation. Another feature of the cycle which makes it complex is that it monitors the process of treatment from the moment the patient is qualified for surgery to one year after surgery. In contrast to the groups of subjects used in other studies, our group was homogenous and consisted of physically active persons, instead of athletes, who are most commonly investigated [29,30].

The purpose of this study was to evaluate the serial change of isokinetic muscle strength around the knee joint before and after ACL reconstruction in males leading a physically active lifestyle and to determine the moment of return to full physical fitness. We hypothesized that six months would not be enough for physically active males to be able to return to the pre-injury activity level, as defined as inter-limb knee torque ratio smaller than 10%. We also hypothesized there would be substantial differences in their muscle torque profiles in consecutive stages of rehabilitation.

## Materials and Methods

### Subjects

The research was conducted on 29 males (age: 27.5 ± 5 years; height: 176.8 ± 5.1 cm; mean body mass measured in four stages of the research: 81.8 ± 10.6 kg) who underwent arthroscopic ACLR after their ACLs had been injured during everyday recreational activity. Prior to the surgery, all the subjects underwent initial rehabilitation to help them regain full knee extension. When they were no longer exhibiting symptoms of pain and inflammation, their orthopaedist made it possible for us to measure their knee joint flexion and extension torques. The subjects were then operated on by the same doctor. The surgeries were performed about 1.5 months after the injury.

The arthroscopic anterior cruciate ligament reconstructions were completed using an anatomic single-bundle technique. The replacement material was four-strand hamstring tendon autografts. The femoral tunnel was created through a single, low anteromedial portal. The surgeon used the Arthrex femoral guide and the remnant preservation technique; ACL fiber remnants within footprint were the landmarks. The insertion “traveled” to the most damaged area, so that as much of the native ACL fiber as possible could be preserved. If there were no remnants, the lateral intercondylar ridge and bifurcate ridge were identified with a shaver and vapor. The single bundle graft was placed to cover as much footprint as possible in relation to both of the bony landmarks.

After the surgery, each patient was provided with an identical protocol for their individual rehabilitation at home. Once a week they saw a physical therapist who planned their exercise program, issued instructions, and corrected their errors in performing the physical exercises. The physical therapy program drew on Shelbourne’s protocol for accelerated rehabilitation after ACL reconstruction [1] and on the most current guidelines [31]. The program is divided into phases based on the stages of tissue recovery (0–2 weeks, 2–4 weeks, 4–8 weeks, 8–12 weeks, and 12–24 weeks). These stages determine the selection of exercises in the closed and open kinematic chain, the period of using a stabilizer and crutches, as well as scar treatment. During the first stages, the program was implemented at the patient’s home (home exercise program, HEP) with the use of such equipment as rubber tapes, balls, or balance discs. Strength training equipment was implemented after the 12^th^ week, when the patient engaged in so-called sports rehabilitation. In our program we did not apply physical therapy procedures such as electro-stimulation, electromagnetic field stimulation, continuous passive motion exercises, or static force training exercises.

Prior to the research, the patients were informed about the purpose of the study and gave their written consent. The research program was approved by the Senate Committee of Scientific Research Ethics at the Josef Pilsudski University of Physical Education in Warsaw.

### Measurements

Knee isokinetic muscle strength was measured at four stages, i.e. prior to the surgery as well as three, six, and twelve months after the reconstruction. They were carried out using a Biodex System 3-PRO (Biodex Medical Systems Inc., Shirley, NY) dynamometer. The subjects performed a 5-minute warm-up on a cycloergometer before the measurements were conducted. Then, they adopted a standard position on a chair and were stabilized with belts, as recommended by the manufacturer of the device. The range of motion (ROM) in the joint was limited to 90 degrees, and the healthy limb was assessed first. The knee flexion and extension torques were evaluated in isokinetic conditions with the commonly used constant angular velocities of 60 deg/s and 180 deg/s [27,32–35]. The test included a series of 5 extending and flexing movements at the velocity of 60 deg/s and 10 attempts at the velocity of 180 deg/s, preceded by three trials with moderate engagement of the muscles. The analysis included only those movements of the extensors and flexors, in which the subjects achieved maximum values of muscle torques. In order to increase the reliability of the measurements, we checked whether the peak torque selected for the analysis did not differ significantly from the remaining ones and we did so immediately after each measurement session using Biodex software. If the selected peak torque was 15% or higher, the test was repeated after a 15-minute break. We checked the reliability of this approach by comparing two maximum values of the torque within each trial. The interclass correlation coefficients computed in this way ranged from 0.96 to 0.98. These values are of the same order of magnitude as those obtained in the test-retest reliability of the Biodex dynamometer ranging between 0.93 and 0.98 at the velocities of 60 deg/s and 180 deg/s [36].

### Data processing

The raw measurement data from the dynamometer were slightly smoothed by means of a fourth-order zero lag Butterworth low-pass filter with a 25 Hz cut-off frequency. This high cut-off frequency was chosen so that possible short disturbances of muscle torques caused by the anterior cruciate ligament injury and by changes in the structure of the knee after the surgery could be recorded. Torque versus position curves were then obtained from the smoothed experimental data. The moment originating from the gravity force affecting the knee attachment of the dynamometer with the calf and foot fastened to it was removed from these characteristics. The removal was possible thanks to the authors’ own scripts which were written in the MATLAB language (MathWorks, Natick, MA), taking into account anthropometric data related to appropriate anatomic segments of the subjects [37] as well as the geometry, mass, and the center of gravity of the attachment. Some of the subjects did not manage to move their limbs within the predefined ROM, even if they had no problems when the ROM was being set up. All the curves were normalized so that they could be compared with one another. The effective ROM was thus replaced by the unit length cycle, and the knee torques were evaluated in 101 evenly distributed points using interpolating cubic splines.

**Fig 1:**
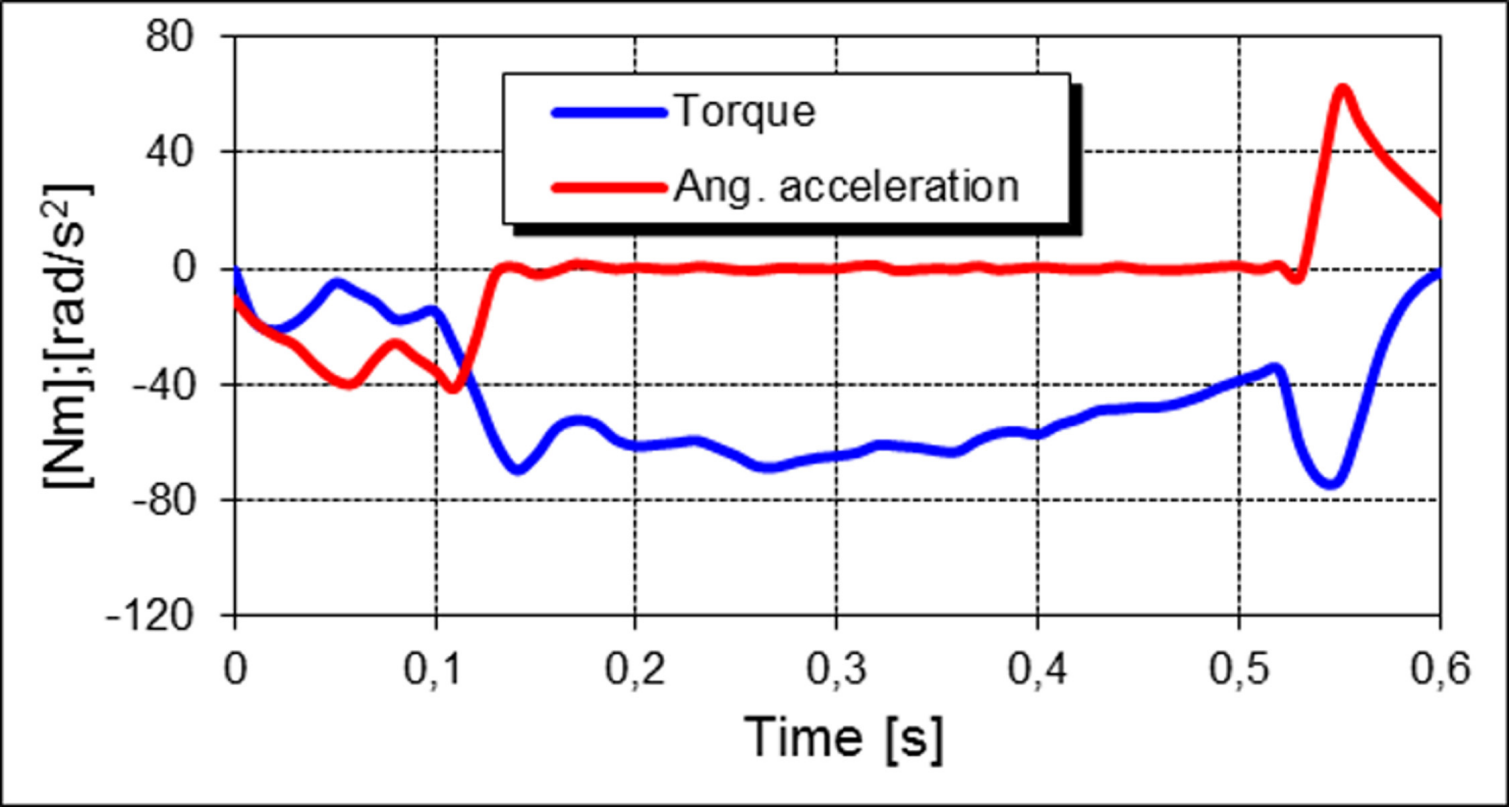
Gravitationally corrected knee flexion torque and angular acceleration of the knee attachment.

At the angular velocity of 180 deg/s, the majority of characteristics, especially those of the knee flexors, are significantly impacted by inertial forces, which occur both in the initial and final phases of the flexing cycle (Fig 1). In the first case, they result from the dynamic action of flexors at the beginning of the movement, while in the second one, they are generated when the lever arm reaches its limit.

In order to choose appropriate peak torques for statistical analysis, the angular velocity time histories from the measurement protocol were differentiated numerically. After the moment of inertia of the attachment was experimentally estimated, inertial effects were calculated for the specified torque-time curves. In the case presented in Fig 1, the value of the knee flexion torque for the abscissa of 0.14 was accepted as the maximum value.

### Statistical analysis

The knee peak torques, H/Q ratios and normalized work of knee muscles were analyzed statistically in order to find significant differences among them at different stages of rehabilitation. Firstly, the experimental/computed data were checked for normality of distribution and homogeneity of variance using the Shapiro-Wilk test and Bartlett’s test. Within-subject limb-to-limb differences and changes over time were then computed by means of a 2-way repeated measures ANOVA with the following design: limb (involved, uninvolved) x time (before surgery and 3 months, 6 months, and 12 months after surgery). One-way ANOVA with repeated measures was applied to detect differences in normalized work at all the stages of rehabilitation. The sphericity assumption between all the pairs of the variables examined was checked using Mauchly’s test. Finally, Bonferroni’s test allowed for a detailed interpretation of significant differences between the mean values of the variables. The level of statistical significance was set at 0.05. The statistical analyses were performed using Statistica 12 (StatSoft, Poland).

## Results

Table 1 shows mean peak torque values for knee extension and flexion at the velocity of 60 deg/s for both the reconstructed (ACLR) and uninvolved limbs in consecutive stages of rehabilitation. In the case of the injured limb, the torque value increased significantly for the extensors (27%) and flexors (18%) between adjoining stages in the period from the 3^rd^ to the 6^th^ month after the operation. Of note is the lack of statistically significant differences between the first and second stages and between the third and fourth stages. No significant differences were identified for the uninvolved limb. The involved/uninvolved peak torque ratio for the knee extensors in the four stages was 0.73 ± 0.19, 0.63 ± 0.18, 0.78 ± 0.16, and 0.85 ± 0.14, respectively. The extensor strength deficit was found to be statistically significant for all of the rehabilitation stages. The involved/uninvolved peak torque ratio for knee flexors was 0.83 ± 0.18, 0.81 ± 0.17, 0.92 ± 0 .11, and 0.96 ± 0.10, respectively. The flexor strength deficits were only statistically significant at the first and second stages of rehabilitation.

**Table 1:** Mean peak torque (Nm) for knee extension and flexion recorded at the velocity of 60 deg/s in consecutive stages of rehabilitation.

	Extension			Flexion		
	ACLR	Uninvolved	Difference	ACLR	Uninvolved	Difference
**Stage 1**	150.99 ± 42.87^4^→	←206.83 ± 45.37	26%	-83.45 ± 20.69^3;4^→	← -100.54 ± 26.19	17%
**Stage 2**	131.06 ± 43.35^3;4^→	←208.00 ± 41.68	37%	-84.01 ± 22.88^3;4^→	← -103.72 ± 23.72	19%
**Stage 3**	166.55 ± 48.11^2^→	←213.50 ± 40.51	22%	-99.25 ± 23.03^1;2^	-107.88 ± 25.03	8%
**Stage 4**	186.80 ± 39.19^1;2^→	←219.84 ± 36.62	15%	-105.99 ± 20.88^1;2^	-110.41 ± 21.75	4%

The upper index indicates a stage with a significantly different (*p* ≤ 0.05) mean peak torque; → ← indicates a significant difference between the two limbs at a particular stage of rehabilitation.

The mean peak torque at the velocity of 180 deg/s was not significantly different between adjoining stages of rehabilitation (Table 2). Neither were significant differences found for the uninvolved limb. The involved/uninvolved peak torque ratio for the knee extensors for the four stages was 0.80 ± 0.23, 0.70 ± 0.21, 0.83 ± 0.16, and 0.89 ± 0.17, respectively. The extensor strength deficit was statistically significant for all stages of rehabilitation. The involved/uninvolved peak torque ratio for the knee flexors amounted to 0.90 ± 0.22, 0.85 ± 0.23, 0.94 ± 0.12, and 0.97 ± 0.13, respectively. The flexor strength deficit was significant at the second stage of rehabilitation only.

**Table 2:** Mean peak torque (Nm) for knee extension and flexion recorded at the velocity of 180 deg/s in consecutive stages of rehabilitation.

	Extension			Flexion		
	ACLR	Uninvolved	Difference	ACLR	Uninvolved	Difference
**Stage 1**	94.69 ± 27.29^4^→	←118.36 ± 29.13	20%	-63.17 ± 22.25^4^	-70.19 ± 20.66	10%
**Stage 2**	92.45 ± 30.35^4^→	←132.07 ± 32.86	30%	-68.19 ± 23.94	-80.22 ± 21.15	15%
**Stage 3**	105.31 ± 32.60→	←126.81 ± 28.13	17%	-76.94 ± 18.83	-81.85 ± 21.12	6%
**Stage 4**	119.01 ± 29.88^1;2^→	←133.71 ±30.03	11%	-80.13 ± 19.99^1^	-82.61 ± 22.56	3%

The upper index indicates a stage with a significantly different (*p* ≤ 0.05) mean peak torque; → ← indicates a significant difference between the two limbs at a particular stage of rehabilitation.

Table 3 presents the H/Q ratio values obtained in the measurements conducted during rehabilitation. This variable is commonly used for interpreting the results of isokinetic measurement. Regardless of the angular velocity, one can observe a substantial increase of the H/Q ratio in the second stage of rehabilitation. There were, however, no statistically significant differences in H/Q ratios between stages. The difference in H/Q ratios between the involved and uninvolved knees was significant at the second stage only.

**Table 3:** H/Q ratio in consecutive stages of rehabilitation.

	Angular velocity 60 deg/s		Angular velocity 180 deg/s	
	ACLR	Uninvolved	ACLR	Uninvolved
**Stage 1**	0.57 ± 0.13	0.50 ± 0.12	0.65 ± 0.15	0.58 ± 0.14
**Stage 2**	0.65 ± 0.15→	←0.51 ± 0.12	0.74 ± 0.19→	←0.61 ± 0.16
**Stage 3**	0.62 ± 0.17	0.52 ± 0.14	0.72 ± 0.14	0.64 ± 0.12
**Stage 4**	0.58 ± 0.13	0.52 ± 0.12	0.68 ± 0.14	0.63 ± 0.13

→ ← indicates a significant difference (*p* ≤ 0.05) between the two limbs at a particular stage of rehabilitation.

**Fig 2:**
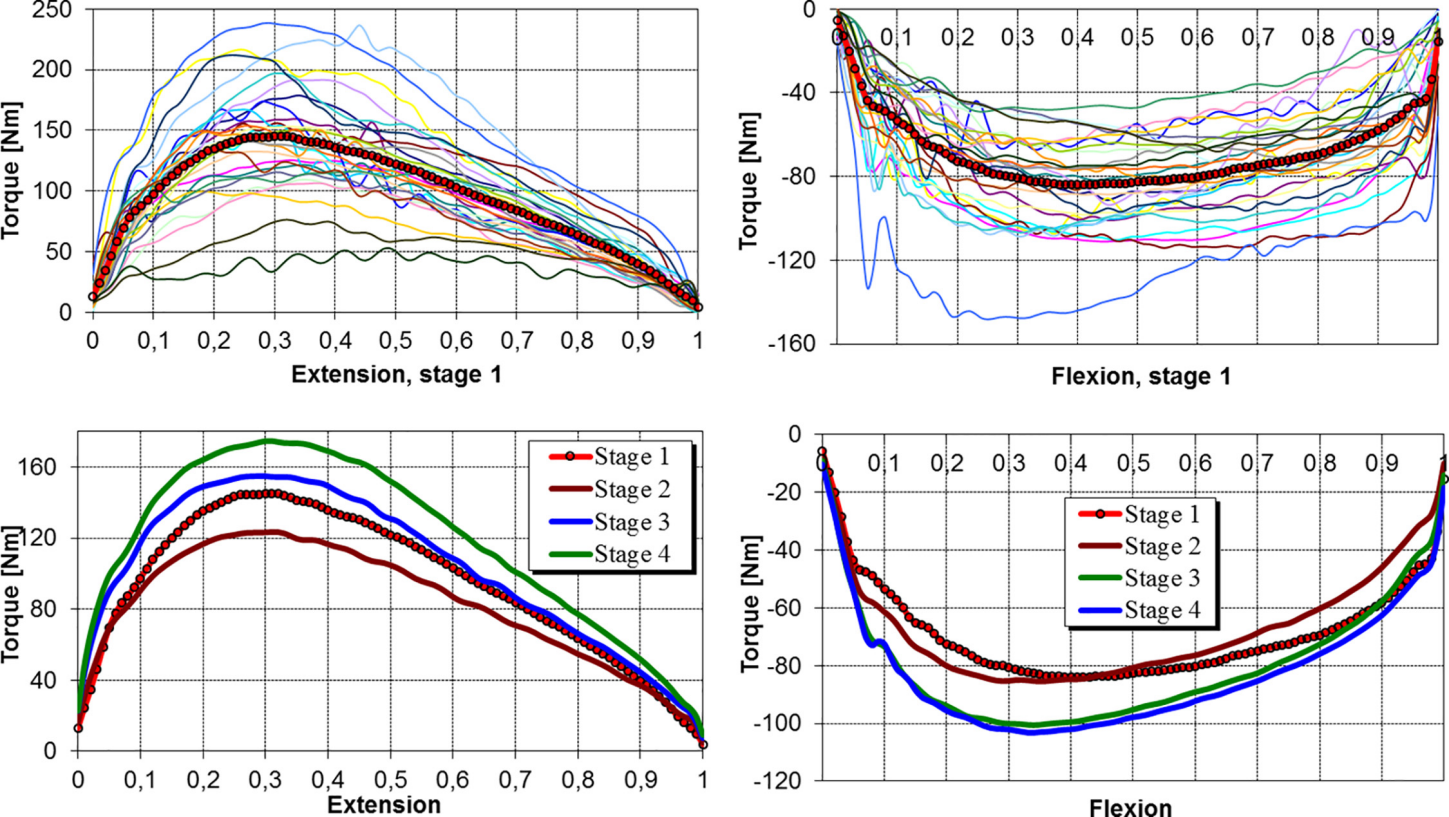
Normalized torque-position characteristics for the involved knee at the angular velocity of 60 deg/s.

Torque-position curves for the involved knee extensors measured at the velocity of 60 deg/s before ACLR was performed are presented in Fig 2 (top left). A thick line marks the resultant curve. A high diversity of peak torques within the range from 50 to 250 Nm is clearly visible. Some of the characteristics have oscillatory patterns in the whole ROM, while others display a distinctive concave profile in the descending phase after reaching the maximum value. Mean torque-position curves of knee extensors computed for particular stages of rehabilitation (Fig 2, bottom left) confirm that torque decreased after the reconstruction, which was followed by a constant increase in torque at all the stages of rehabilitation. Peak torques were identified at one-third of the ROM, irrespective of the rehabilitation stage. Due to the significant variability of these values, the rate of increase and decrease of the extension torque is different, particularly between the second and fourth stages.

The torque-position characteristics of the involved knee flexors measured at the velocity of 60 deg/s before ACLR and depicted in Fig 2 (top right) indicate a significant diversity of these characteristics. Their peak values cover the range from -40 to -140 Nm. Some of the curves have oscillatory patterns in the whole ROM, as was the case with the extensors. Local extremes of the moments, which originate from inertial forces, are discernible in the initial phase of the movement, between the 0.1 and 0.2 parts of the ROM, as well as in the final one. Slight differences in peak flexion torques (Fig 2, bottom right) can be observed between the first two stages and between the last two stages of rehabilitation, whereas a significant increase in peak torque was noted between the first two stages and stage 3.

Fig 2 (bottom right) shows that the rate of increase and decrease of mean normalized knee flexion torque also depends on the stage of rehabilitation and changes over the period of one year. Contrary to the previously presented characteristics of the extensors, a clear shift of peak torque to the right can be observed after the first stage of rehabilitation. A similarity between flexion torque characteristics may be observed in the first two stages, highlighting the fact that the strength of the flexors was not affected despite the surgical intervention that was performed when the graft was being prepared.

**Fig 3:**
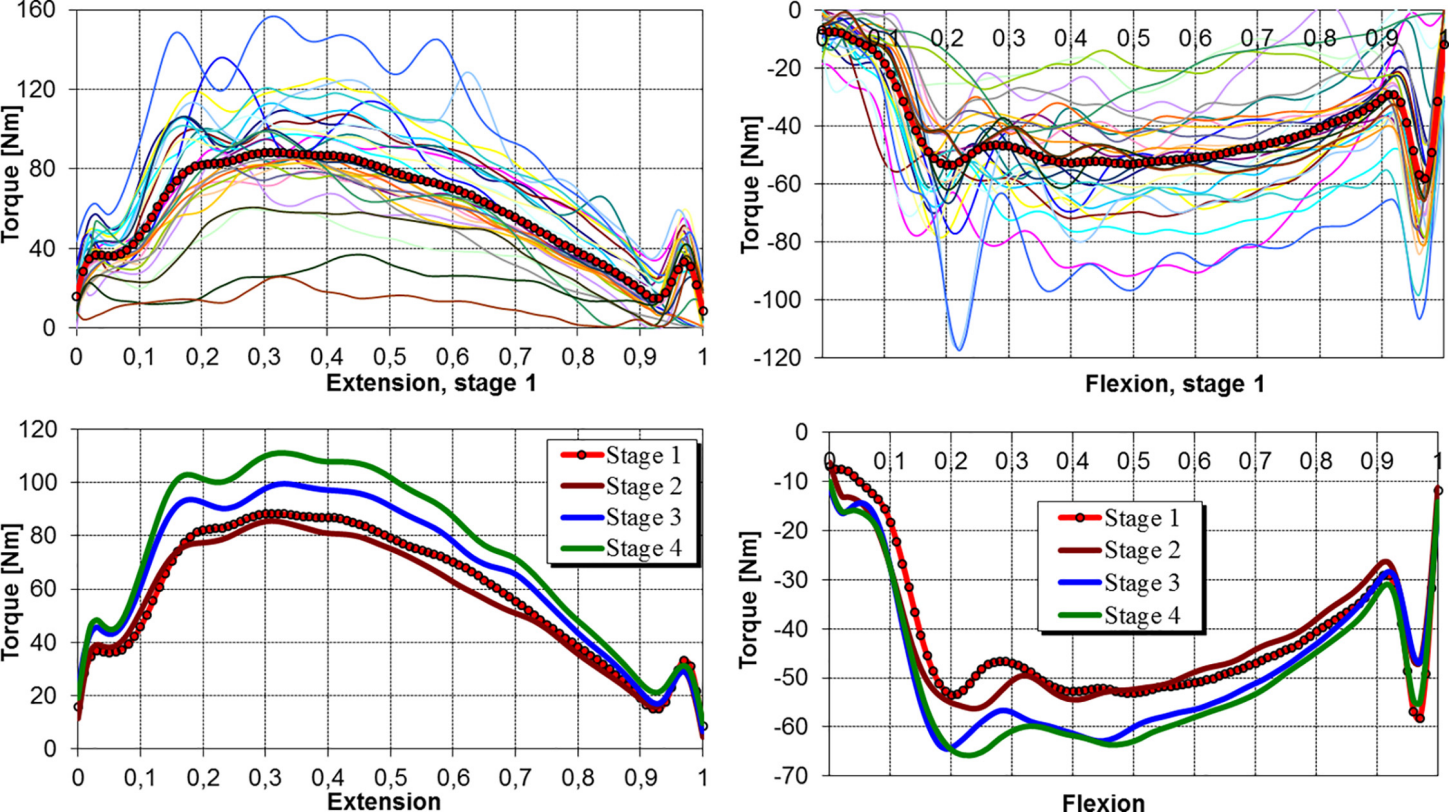
Normalized torque-position characteristics for the involved knee at the angular velocity of 180 deg/s.

The torque-position curves of the involved knee extensors recorded at the velocity of 180 deg/s before ACLR are illustrated in Fig 3 (top left). What should be emphasized is the vast diversity of peak torques which achieve values falling within the range from 25 Nm to 160 Nm. Again, one can observe the influence of inertial forces in the form of local peaks in the initial and final parts of the cycle as well as a more oscillating character of the curves compared to the ones obtained at the velocity of 60 deg/s. The mean torque-position curves of the knee extensors (Fig 3, bottom left) clearly indicate that the largest increase in torque value occurs during the 3^rd^ stage. The rate of increase and decrease of the torque is different, particularly in the first and fourth stages. Two local extremes at the beginning and at the end of the ROM confirmed the influence of inertial forces in the initial and final phases of the movement. The characteristics did not show a clear decrease in the value of the torque between the first and second stages similar to the one observed at the velocity of 60 deg/s. This means that the reduction of the strength of the quadriceps occurring in this period was not identified at the velocity of 180 deg/s.

The torque vs. position characteristics of the knee flexors recorded at the velocity of 180 deg/s before ACLR are illustrated in Fig 3 (top, right). Almost all the curves contain considerable oscillations with a larger frequency and amplitude than in the case of the extensors examined at the same velocity. Flexion peak torques fall within the range from -20 Nm to -120 Nm. In the final phase of the flexing cycle, the influence of inertial forces is also evident. Fig 3 (bottom right) presents the mean torque vs. position characteristics of the knee flexors measured in particular stages of rehabilitation. Slight differences are noticeable between the curves of the last two stages of rehabilitation, and a significant increase in torque values in the period from the 3^rd^ to 6^th^ month after the reconstruction can be discerned.

Additional possibilities of quantitative assessment arise if one examines the areas below the curves (Figs 2 and 3), since they can be interpreted as the normalized work of the muscles. Mean values of this variable are presented in Table 4. The statistical analysis of normalized work generally confirmed the significance of the differences previously noted for peak torques, except for the differences between the first and third stages at the velocity of 60 deg/s (*p* ≤ 0.13) and between the first and fourth stages at the higher velocity (*p* ≤ 0.12) for the knee flexors.

**Table 4:** Normalized work (Nm) of the involved leg in consecutive stages of rehabilitation.

	Angular velocity 60 deg/s		Angular velocity 180 deg/s	
	Extension	Flexion	Extension	Flexion
**Stage 1**	94.90 ± 26.73^4^	68.65 ± 19.12^4^	59.70 ± 19.11^4^	42.35 ± 13.35
**Stage 2**	83.23 ± 28.09^3;4^	66.92 ± 20.11^3;4^	57.03 ± 19.30^4^	42.77 ± 16.58
**Stage 3**	104.70 ± 28.95^2^	79.59 ± 17.91^2^	68.16 ± 21.88	48.13 ± 11.45
**Stage 4**	118.81 ± 24.91^1;2^	82.24 ± 16.55^1;2^	75.51 ± 18.02^1;2^	50.01 ± 11.01

The upper index indicates a stage with significantly different (*p* ≤ 0.05) mean normalized work.

## Discussion

The main aim of this study was to evaluate the serial change of isokinetic muscle strength around the knee joint before and after ACL reconstruction. We identified extension and flexion peak torques, as well as calculated the uninvolved/involved peak torque ratio for the knee extensors and flexors, computed the H/Q ratio, and determined the torque-position characteristics of these muscles at 4 stages of rehabilitation.

Mean peak torques at the velocity of 60 deg/s of both muscle groups increased significantly in the period between the 3^rd^ and 6^th^ months after the reconstruction, indicating the importance of this strength-oriented phase of rehabilitation. The lack of significant differences between the first and second stages can be explained by a wide spread of the measurement data caused by the possible presence of some non-copers among the subjects [38]. Regardless of the angular velocity, the strength of the extensors improved continually between the third and fourth stages, although the differences were statistically insignificant and the rate of change in strength was lower than in the preceding stage. This is probably due to the fact that up to the third stage of the rehabilitation the patients underwent intensive therapeutic training, whereas the time assigned for exercise was significantly reduced when the treatment was completed after 6 months.

The values of peak torques obtained in our research are relatively similar to those presented in the work of Karnikas et al. [33]. The slightly higher values of extension torques recorded by us mainly stem from the fact that, in contrast to the above-mentioned study where all three groups of subjects included women, all of our subjects were male. The lower values of flexion torques computed in our study are due to the fact that Karnikas and his colleagues probably did not apply the gravity correction, though they did not report on this explicitly. The application of this correction has a considerable impact on the results of such studies; for instance, our calculations showed, that flexion peak torque at the angular velocity of 60 deg/s before ACLR was even 12% lower than that recorded without gravity correction.

The involved/uninvolved peak torque ratio was the second variable to be analyzed. This index is perceived as an indicator of the symmetry of muscle strength in the two limbs. Its value, expressed as a percentage difference, should not exceed ± 10% for healthy limbs. Differences higher than 20% indicate a pathological state, while those falling within the range <10÷20>% signal the risk that such a state has developed [39]. Our results indicated that the involved/uninvolved torque ratio evolved during the rehabilitation. It achieved its minimum value after the reconstruction and increased successively. The largest deficit, amounting to 37%, was recognized for the knee extensors at the velocity of 60 deg/s. The injured limb did not achieve the level of strength of the healthy one in any of the analyzed stages. Moreover, irrespective of the velocity, the differences between extension peak torques remained significant at all stages. As far as the results of similar studies are concerned, Keays et al. [40] reported differences in the strength of the knee extensors at the level of 12% six months after the reconstruction. Yasuda et al. [41] estimated that the percentage difference between peak torque of the involved and uninvolved quadriceps at the velocity of 60 deg/s amounted to 17% one year after the surgery, whereas 18 months after the reconstruction the strength deficit between the two limbs was evaluated at 11% [35].

The H/Q ratio was another variable which was examined. The value of this ratio depends on many factors, including the angular velocity used, the patients’ position during testing, as well as their age and gender. The values of this ratio for a healthy limb therefore have a fairly wide range, from 0.43 to 0.9 [42]. An H/Q ratio of 0.61 at the velocity of 60 deg/s and that of 0.72 at the velocity of 180 deg/s are usually regarded as normative data [43], although the variability of these coefficients was not provided by the Biodex manufacturer. In our study, the largest value of this ratio for the involved limb was found 3 months after surgery. Regardless of the velocity, significant differences between the involved and healthy limbs were also present at this stage. The average H/Q ratios for the healthy limb were always lower than the normative data. This observation suggests that the H/Q ratio for an involved limb should be compared to the H/Q ratio of the healthy limb instead of normative data. A similar conclusion was made by Kannus [44], who examined lower extremities with knee joint dysfunction caused by damage to the ACL.

Determining mean normalized torque vs. position curves of the knee extensors and flexors made it possible to carry out both qualitative and quantitative analyses of these characteristics. The qualitative analysis, which is presented in the preceding section, highlighted substantial differences in muscle torque profiles in consecutive stages of rehabilitation. In order to perform a quantitative interpretation of these curves, the extension torque characteristics obtained at the velocity of 60 deg/s were smoothed using cubic splines. The first derivatives were then calculated for the point with an abscissa of 0.1. This point corresponds to its equivalent on the torque-time curve at 0.18 s and is significant from a clinical perspective [43]. As a result, it was concluded that the percentage differences between adjoining stages reached the levels of -19%, 31%, and 7%. The minus sign indicates a relative decline of the slope of the curve. A similar procedure may be carried out for other essential points of the ROM. The other method of quantitative assessment ensures the normalized work of the muscles. As mentioned earlier, the statistical analysis of this variable generally confirmed the results obtained in the analysis of peak torques. It is worth emphasizing that normalized work used as a dependent variable in ANOVA was more robust in meeting the sphericity condition than peak torques.

The second aim of this study was to determine the moment of safe return to full physical fitness for males having an active lifestyle. Significant limb asymmetry in extension peak torques (of 28.2% and 20.5% assessed at the velocities of 60 deg/s and 180 deg/s, respectively) in the third stage means that a period of 6 months is insufficient for the recovery of a knee joint to the level prior to ACLR. A similar conclusion can be drawn regarding the physical fitness of the subjects 12 months after the surgery, when limb asymmetry was still at the level of 17.6% and 12.4%, which is higher than the 15% deficit norm defined in [26] or the more conservative 10% threshold proposed in [39]. These findings are in line with the work of Ardern et al. [29], who estimated the rate of return to competitive sport among athletes to be 33% twelve months after ACLR.

The results of biomechanical measurements are also of clinical relevance, since they helped to assess the progress of the rehabilitation and to implement necessary modifications aimed at optimizing strength or endurance training loads for particular groups of muscles. Six months after ACL reconstruction, the results of biomechanical measurements appeared to be crucial when it came to the doctor’s statement concerning the completion of the training treatment and recommendations for further rehabilitation.

While analyzing the results obtained in this study, the importance of at least two factors which could limit the significance of these results should be emphasized. The first one concerns the inaccuracy of the measurement of knee extensor and flexor torques in isokinetic conditions. Despite eliminating the influence of the force of gravity and removing inertial force effects when using peak values for statistical purposes, the errors resulting from the lack of collinearity of the axis of rotation in the knee joint with the axis of the dynamometer shaft were not corrected [34,45–46]. These errors, expressed in the form of the differences between the real value for joint angle and the value measured by the dynamometer while extending the knee at the velocity of 60 deg/s, reached the level of 13±2 deg [34]. When expressed as a difference between the real value of the moment and the value registered by the dynamometer, the errors were estimated at the level of 3.5–7.3% [45]. Removing these errors is practically impossible because of the relative movement of the limb with respect to the knee attachment.

Another factor concerns the healthy limb served as a reference point for the results of the measurement of the injured limb. The strength of this limb changed during rehabilitation, which also influenced the conclusions drawn from the measurements. Despite this inconvenience, this procedure is commonly accepted in biomechanical analyses, and convincing arguments supporting such an approach have been discussed by Scanlan et al. [13].

## Conclusions

Evaluating the serial change of isokinetic muscle strength around the knee joint before and after ACL reconstruction according to the proposed schedule made it possible to assess the dynamics of the rehabilitation process.

One year after anterior cruciate ligament reconstruction may be too early to return to full physical fitness for males who are physically active.

The strength of the knee flexors and extensors was estimated at two different angular velocities. The conclusions drawn from the analysis at the velocity of 60 deg/s do not fully comply with those stemming from the results obtained at the velocity of 180 deg/s.

The research was carried out in a homogeneous group as far as age and gender are concerned. The findings may thus constitute useful referential data for individuals coming from similar populations.
